# Voxel-based morphometry of grey matter structures in Parkinson’s Disease with wearing-off

**DOI:** 10.1007/s11682-023-00793-3

**Published:** 2023-09-22

**Authors:** Heng Zhai, Wenliang Fan, Yan Xiao, Zhipeng Zhu, Ying Ding, Chentao He, Wei Zhang, Yan Xu, Yuhu Zhang

**Affiliations:** 1 Department of Neurology, Guangdong Neuroscience Institute, Guangdong Provincial People’s Hospital, Guangdong Academy of Medical Sciences, Southern Medical University, No. 106 Zhongshan Er Road, Guangzhou, 510080 Guangdong Province China; 2 Department of Neurology, Union Hospital, Tongji Medical College, Huazhong University of Science and Technology, Wuhan, 430022 Hubei Province China; 3 Department of Radiology, Union Hospital, Tongji Medical College, Huazhong University of Science and Technology, Wuhan, 430022 Hubei Province China; 4 Hubei Province Key Laboratory of Molecular Imaging, Wuhan, 430022 Hubei Province China; 5 Guangzhou Key Laboratory of Diagnosis and Treatment for Neurodegenerative Diseases, Guangdong Provincial People’s Hospital, Guangdong Academy of Medical Sciences, Guangzhou, 510080 China; 6 Guangdong Provincial Key Laboratory of Artificial Intelligence in Medical Image Analysis and Application, Guangdong Provincial People’s Hospital, Guangdong Academy of Medical Sciences, Guangzhou, 510080 China

**Keywords:** Parkinson’s Disease, Wearing-off, Grey matter, Voxel-based morphometry

## Abstract

Our study aimed to investigate the grey matter (GM) changes using voxel-based morphometry (VBM) in Parkinson’s disease (PD) patients with wearing-off (WO). 3D-T1-weighted imaging was performed on 48 PD patients without wearing-off (PD-nWO), 39 PD patients with wearing-off (PD-WO) and 47 age and sex-matched healthy controls (HCs). 3D structural images were analyzed by VBM procedure with Statistical Parametric Mapping (SPM12) to detect grey matter volume. Widespread areas of grey matter changes were found in patients among three groups (in bilateral frontal, temporal lobes, lingual gyrus, inferior occipital gyrus, right precuneus, right superior parietal gyrus and right cerebellum). Grey matter reductions were found in frontal lobe (right middle frontal gyrus, superior frontal gyrus and precentral gyrus), right parietal lobe (precuneus, superior parietal gyrus, postcentral gyrus), right temporal lobe (superior temporal gyrus, middle temporal gyrus), bilateral lingual gyrus and inferior occipital gyrus in PD-WO group compared with the PD-nWO group. Our results suggesting that wearing-off may be associated with grey matter atrophy in the cortical areas. These findings may aid in a better understanding of the brain degeneration process in PD with wearing-off.

## Introduction

Parkinson’s disease (PD) is a chronic and progressive neurodegenerative disease characterized by both motor and nonmotor symptoms. The pathophysiology of PD is loss of the dopaminergic neurons in the substantia nigra and development of Lewy Bodies (Cacabelos, 2017). The chronic administration of dopaminergic therapy induces the “wearing-off phenomenon” (Pistacchi et al., 2017). However, the incidence of wearing-off remains under-recognized because of the heterogeneity of patients. The reported prevalence of wearing-off (WO) showed a wide range (from 12 to 60%) in PD patients within 4–6 years of dopaminergic therapy (Chen et al., 2015; Warren et al., 2013). According to Braak et al. hypothesis, Lewy body starting from the brain stem and progressing affects the neocortex in PD (Braak et al., 2004). However, the exact morphological changes in the brain of wearing-off remain unclear.

Voxel-based morphometry (VBM) is an automated quantitative magnetic resonance imaging (MRI) technique extensively used to assess the grey matter (GM) morphology changes in the brain and has been widely used in PD (Li et al., 2017; Pan et al., 2013). The majority of the studies focused on grey matter (GM) changes in PD associated with motor and nonmotor symptoms. Some studies showed that PD patients with mild cognitive impairment (MCI) have an atrophy in GM in temporal, hippocampus and limbic regions (Rektorova et al., 2014; Zhang et al., 2015). Dyskinesia patients with PD present with increased GM volume of the inferior frontal cortex than those without dyskinesia (Cerasa et al., 2011). To the best of our knowledge, the present study was the first to investigate alterations in the GM volume of the brain in PD patients with wearing-off.

We hypothesized that patients with wearing-off would have more cortical atrophy than those without wearing-off. This study aimed to examine the structural brain changes in PD patients with wearing-off based on VBM analysis. It is hoped that this study will help us better understand the pathophysiological mechanism in the PD patients with WO.

## Methods

### Patients

This study was approved by the Ethics Committee of Union Hospital, Tongji Medical College of Huazhong University of Science and Technology and Guangdong Provincial People’s Hospital. Informed consent was obtained from all the subjects. The study was also conducted in accordance with the Declaration of Helsinki. Eighty-seven patients with PD were recruited between September 2020 and May 2022. Forty-seven healthy controls (HCs) matched by age, gender and education level were selected. The inclusion criteria were as follows: 1) patients diagnosed with PD according to the 2015 Movement Disorder Society clinical diagnostic criteria (Postuma et al., 2015); 2) right-handed Chinese natives; 3) received levodopa and/or dopamine agonist (DA) therapy for 6 months or longer; 4) patients without dyskinesias; and 5) patients whose Parkinsonism was induced by cerebrovascular disease, medications, encephalitis, poisoning, trauma and other neurodegenerative diseases were excluded. HCs were included without any neurological disease such as stroke, brain tumor, severe mental disorders, or white matter lesions more than 1 mm visible on structural MRI.

Baseline demographic and clinical data including age, gender, education level, disease duration, Hoehn and Yahr Scale (H&Y), Unified Parkinson’s Disease Rating Scale (UPDRS) (Goetz et al., 2008) score and use of anti-Parkinson medication were recorded. Neurological examinations of cognitive and affective disorder states were evaluated by the Mini-Mental State Examination (MMSE) (Katzman et al., 1988), Montreal Cognitive Assessment (MoCA) (Lu et al., 2011), Hamilton Rating Scale for Depression (HAMD)(Hamilton, 1960) and Hamilton Rating Scale for Anxiety (HAMA)(Hamilton, 1959). When the education years of the subjects were less than 12 years, 1 score was added on their MoCA total score (Nasreddine et al., 2005). For PD patients, the third part of the UPDRS (UPDRS-III) was used to assess the severity of motor symptoms and evaluated in the daytime during their ON (approximately 1 h after the dose of medication) and OFF (approximately 12 h after the dose of medication) phases (Si et al., 2022). All PD patients were receiving stable dopaminergic treatment prior to the assessment. The levodopa equivalent daily dose (LEDD) was calculated for each PD patient (Tomlinson et al., 2010). The validated Chinese version of the 9-item Wearing-off Questionnaire (WOQ-9) was applied for screening wearing-off, and at least one improved symptom after next dose of medication indicated a diagnosis of WO (Chan et al., 2011). The daily OFF time of the WO group was also assessed using UPDRS item 4.3. Then PD patients were classified into two groups: patients without wearing-off (PD-nWO) and patients with wearing-off (PD-WO).

### Magnetic resonance imaging acquisition

All patients were performed on a 3.0 Tesla MRI scanner (MAGNETOM Skyra; Siemens Healthcare, Erlangen, Germany) using a 20-channel head/neck coil. All participants were required to lay still in the supine position. Ear-plugs were used to reduce the large noise made by the scanner, and tight foam padding was used to minimize head motion. After an overnight withdrawal of dopaminergic medications for 12 h prior to the MRI scan. Structural three-dimensional T1-weighted images (3D-T1WI) were obtained using a volumetric 3D magnetization-prepared rapid gradient-echo (MP-RAGE) sequence with following parameters: repetition time (TR) = 2400 ms, echo time (TE) = 2.26 ms, flip angle (FA) = 8°, field of view (FOV) = 256 × 256 mm^2^, voxel size = 1 × 1 × 1 mm^3^, slice thickness = 1 mm, slice number = 192, and matrix size = 256 × 256.

### Magnetic resonance imaging data processing

Preprocessing of the high-resolution T1-weighted structural images was analyzed for VBM using the CAT12 toolbox within the Statistical Parametric Mapping (SPM12, Wellcome Department of Imaging Neuroscience Group; http://www.fil.ion.ucl.ac.uk/spm.fil.ion.ucl.ac.uk/spm) in MATLAB software. The anatomical images were first segmented into GM, white matter (WM), and cerebrospinal fluid (CSF) using the unified segmentation module (Ashburner & Friston, 2005). Then segmented GM images were normalized into the Montreal Neurological Institute (MNI) standard space using the diffeomorphic anatomic registration through exponentiated lie algebra algorithm (DARTEL) (Ashburner, 2007) tool. After affine and nonlinear registration of the GM templates in MNI space, images were then modulated to ensure that relative GM volumes were preserved following the spatial normalization process. Finally, the resulting GM images were smoothed with a 10 mm full-width-at-half-maximum (FWHM) Gaussian kernel.

### Statistical analysis

Statistical analysis was performed using Statistic Package for Social Science (SPSS) software version 23.0. Continuous variables are presented as the means and standard deviations. Categorical variables were expressed as counts and percentages. Independent samples *t* test or Mann–Whitney U test was applied to compare two groups. The chi-squared test was used to compare categorical variables. One-way analysis of variance (ANOVA) was used to compare GM volume differences among the HC, PD-nWO, and PD-WO groups after controlling for age, gender, education level, HAMA, HAMD, MMSE, and MoCA scores (*P* < 0.05). A post hoc Tukey pairwise comparison was performed to compare the GM volume changes between HC and PD-nWO, HC and PD-WO, and between PD-nWO and PD-WO. The family-wise error (FWE) correction was performed for multiple comparisons with a confidence threshold of *P*_FWE_ < 0.05. The cluster size of 10 voxels extent threshold was chosen. Finally, we used xjview (2021) toolbox to record voxel area (represented with pseudo color), with activation volume (cluster size threshold of 10 voxels), activation intensity (statistically analyzed with t-test and expressed as F value; F value is proportional to the intensity). Spearman correlation analyses were performed to assess relationships between the regional GM volume and clinical variables (UPDRS-III, MMSE, MoCA, HAMA and HAMD scores) of PD patients with wearing-off with age, gender and education level as covariates. A *P*-value less than 0.05 was considered statistically significant.

## Results

### Characteristics of the patients

A total of 48 PD-nWO patients, 39 PD-WO patients, and 47 healthy controls were eventually included in the analysis. The demographic and clinical characteristics of the three groups are listed in Table 1. No significant differences were observed in age, gender, or education level between the HC, PD-nWO and PD-WO groups (*P* > 0.05). The mean age of the PD-WO, PD-nWO and HC groups were 66.38 ± 9.33, 66.90 ± 8.57 and 65.62 ± 6.79 (mean ± SD) years, respectively (*P* = 0.749). In the PD patients with WO, the mean onset age was younger (62.46 ± 9.40 versus 64.64 ± 9.07 years), with a longer disease duration (3.79 ± 2.40 versus 2.78 ± 1.88 years), while the differences were not significant (*P* > 0.05). The PD-WO group had higher H&Y score (2.77 ± 0.97 versus 1.54 ± 0.51) than those without WO (*P* < 0.01). In terms of cognitive performance, patients with WO had poorer scores measured with the MMSE and MoCA scores than patients without WO or HC subjects (*P* < 0.01). The scores of HAMA and HAMD in PD patients with and without WO were significantly higher than those in healthy controls (*P* < 0.01). In addition, patients with WO had higher UPDRS-III scores (37.49 ± 14.72 versus 23.02 ± 11.96) than those without WO (*P* < 0.01).

**Table 1:** Clinical and demographic characteristics of HC, PD-nWO and PD-WO

	HC	PD-nWO	PD-WO	*P*-value
	*n* = 47	*n* = 48	*n* = 39	
Age (years)	65.62 ± 6.79	66.90 ± 8.57	66.38 ± 9.33	0.749^a^
Gender (male/female)	15/32	21/27	16/23	0.469^b^
Education level (years)	8.28 ± 4.31	8.40 ± 4.17	7.90 ± 4.53	0.859^a^
Age at symptom onset (years)	N/A	64.64 ± 9.07	62.46 ± 9.40	0.344^c^*
Disease duration (years)	N/A	2.78 ± 1.88	3.79 ± 2.40	0.075^d^*
Hoehn & Yahr stage	N/A	1.54 ± 0.51	2.77 ± 0.97	0.000^d^*
UPDRS part I score	N/A	6.10 ± 4.54	7.38 ± 4.21	0.180^d^
UPDRS part II score	N/A	7.85 ± 6.23	14.00 ± 8.78	0.000^c^*
UPDRS part IIII score	N/A	23.02 ± 11.96	37.49 ± 14.72	0.000^c^*
MMSE scores	26.51 ± 2.03	25.69 ± 3.20	22.64 ± 5.80^23^	0.000^a^*
MoCA scores	22.38 ± 3.25	21 ± 5.27	18.49 ± 5.81^23^	0.000^a^*
HAMA scores	4.49 ± 4.75	9.35 ± 8.80^1^	11.23 ± 8.93^2^	0.000^a^*
HAMD scores	7.02 ± 8.22	11.71 ± 10.19^1^	15.460 ± 7.62^2^	0.000^a^*

Data are expressed as mean ± standard deviation or number

*HC*, healthy controls; *PD*, Parkinson’s disease; *WO*, Wearing-off; *UPDRS*, Unified Parkinson’s Disease Rating Scale; *WOQ-9*, WEARING OFF Questionnaire 9 items; *MMSE*, Mini-Mental State Examination; *MoCA*, montreal cognitive assessment; *HAMA*, Hamilton Anxiety Scale; HAMD, Hamilton Depression Scale; LEDD, levodopa equivalent daily dose

*P < 0.05 was considered significant differences

^a^*P* Values are from One-way ANOVA. Post-hoc comparisons further revealed the source of ANOVA derived differences (1: PD-nWO vs HC; 2: PD-WO vs HC; 3: PD-nWO vs PD-WO)

^b^*P* Values are from Chi-squared test

^c^*P* values are from Two-sample t-test

^d^*P* values are from Mann–Whitney U test

### Comparison of GM changes among three groups

**Fig. 1:**
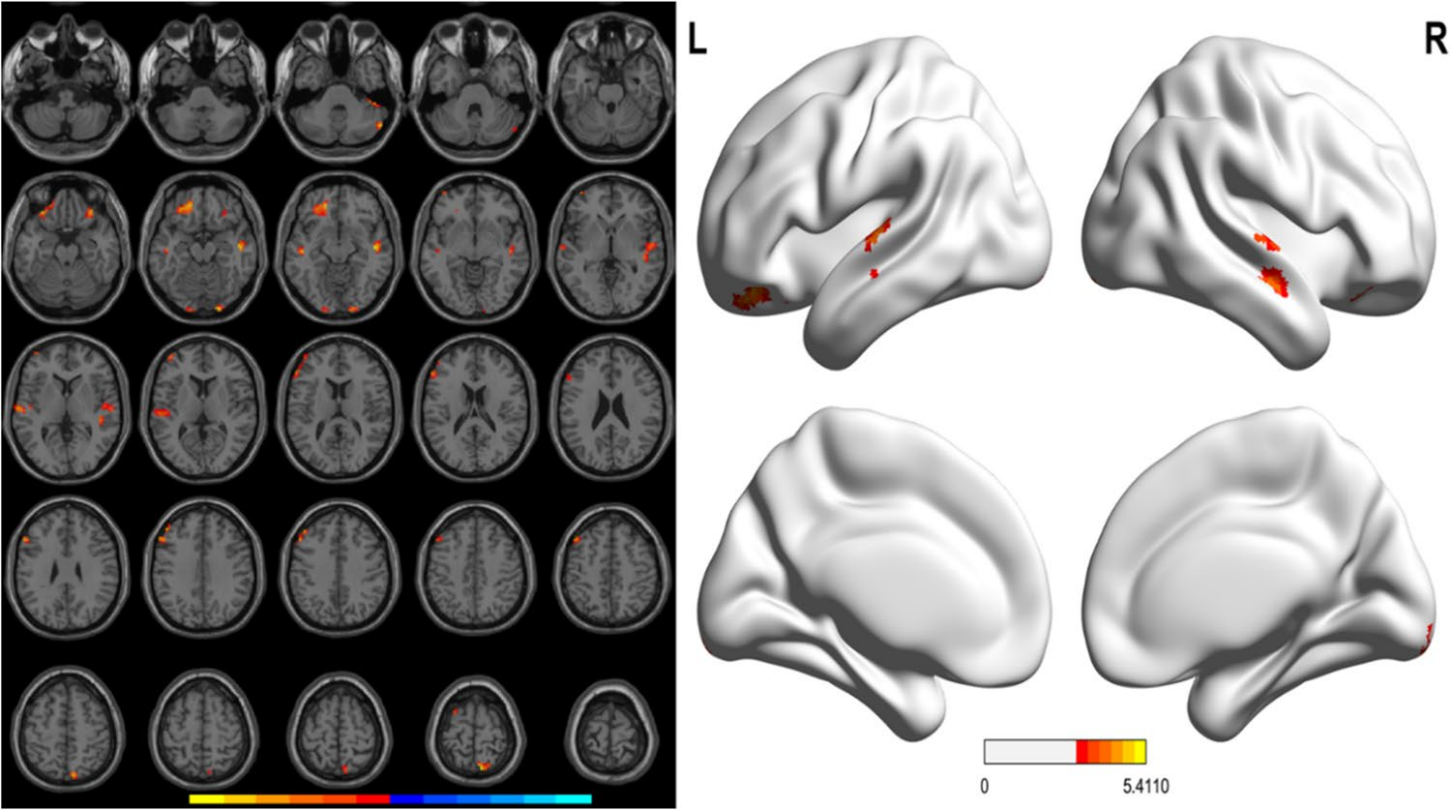
Significant differences in GM volumes among the HC, PD-nWO and PD-WO groups. Correction for multiple comparisons (FWE, *P* < 0.05) was used to threshold the analysis. The color bar represents the F score. Yellow presents a high F score. Abbreviations: HC, healthy control; PD, Parkinson’s disease; WO, wearing-off; GM, grey matter; L, left, R, right

Whole brain VBM analysis among the three groups revealed significant differences in GM volume in the following regions: right cerebellum (right cerebellum Crus I, posterior cerebellar gyrus), bilateral frontal cortex (left middle frontal gyrus, bilateral orbital part of middle frontal gyrus, left orbital part of superior frontal gyrus, left orbital part of inferior frontal gyrus, right orbital part of superior frontal gyrus, left triangular and opercular part of inferior frontal gyrus, and left dorsolateral superior frontal gyrus), bilateral temporal cortex (superior temporal gyrus, middle temporal gyrus, and right Heschl’s gyrus), bilateral precuneus, superior parietal gyrus, lingual gyrus and inferior occipital gyrus. These findings were reported in Table 2 and Fig. 1.

**Table 2:** Differences in grey matter volume among HC group, PD-nWO group and PD-WO group

Cluster	L/R	Anatomical region	Voxels	Peak MNI coordinates			*F* value
				X	Y	Z	
1	R	Cerebellar Crus I	10	48	−36	−36	4.2575
2	R	Cerebellar Crus I, cerebellar posterior lobe	20	54	−66	−33	4.8195
3	L	Middle frontal gyrus, orbito-frontal gyrus	124	−33	33	−21	4.8418
4	R	Orbital of middle frontal gyrus, medial orbital of inferior frontal gyrus	27	30	33	−21	4.4456
5	R	Medial temporal gyrus, superior temporal gyrus, Heschl’s gyrus	114	48	−15	−12	5.411
6	R	Lingual gyrus, inferior occipital gyrus	20	21	−96	−15	4.9233
7	L	Inferior occipital gyrus, lingual gyrus	12	−18	−99	−12	3.606
8	L	Medial temporal gyrus	19	−48	−21	−12	4.4374
9	L	Middle frontal gyrus	27	−42	60	−3	4.375
10	L	Medial temporal gyrus, superior temporal gyrus, Heschl’s gyrus	60	−63	−15	3	4.4923
11	L	Middle frontal gyrus, triangular inferior frontal gyrus, opercular part of inferior frontal gyrus	111	−48	36	36	5.137
12	R	Precuneus, superior parietal gyrus	56	9	−69	69	5.0975
13	L	Dorsolateral of superior frontal gyrus	12	−27	9	69	3.6824

MNI, Montreal Neurological Institute space; The X, Y, and Z coordinates refer to the anatomical location of brain areas

*PD*, Parkinson's disease; *HC*, healthy control group; *WO*, wearing-off; *L*, left; *R*, right

### Comparison of GM changes between PD-nWO and HC groups

**Fig. 2:**
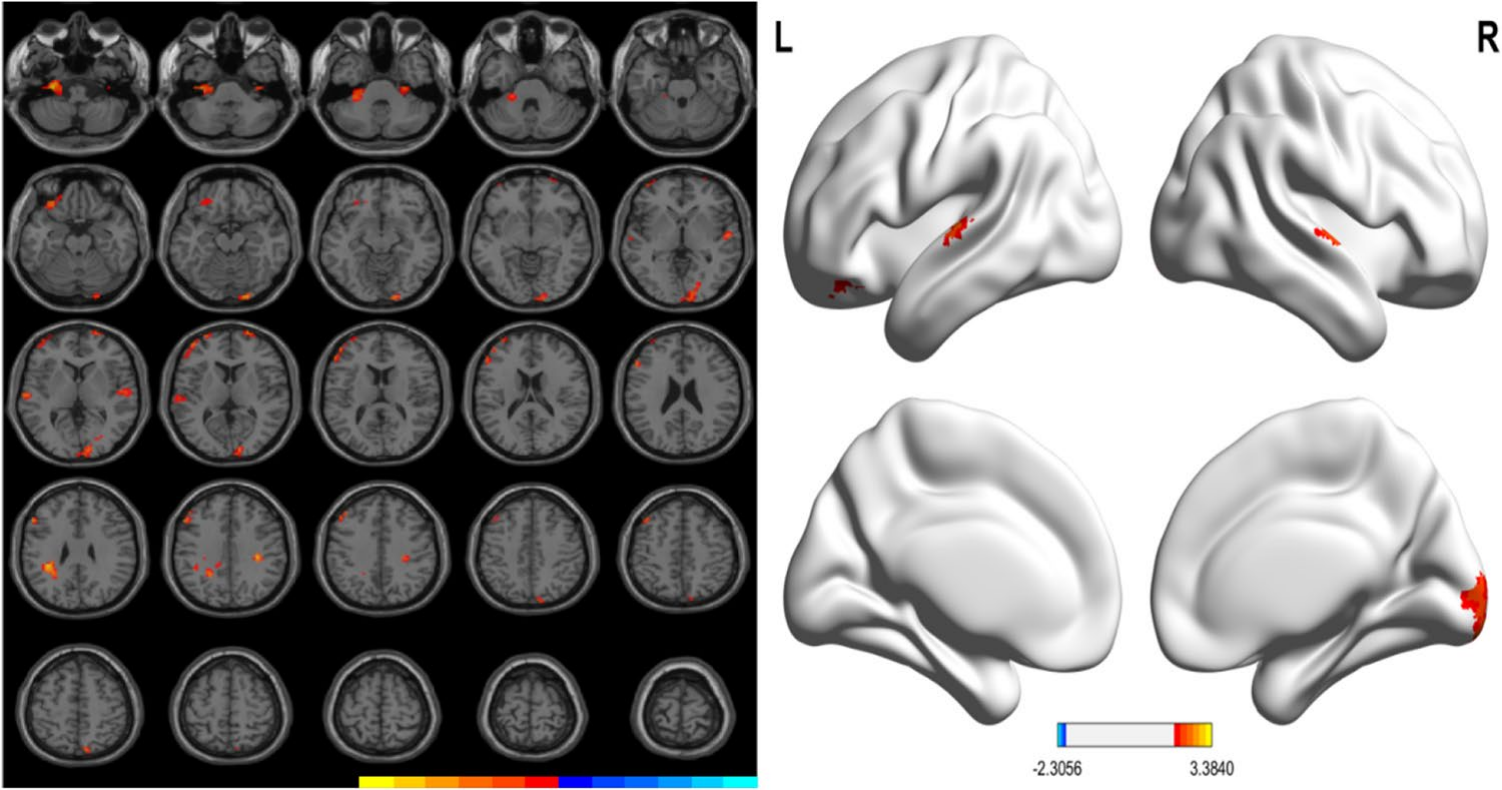
Significant differences in GM volumes between the HC and PD-nWO groups. Correction for multiple comparisons (FWE, *P* < 0.05) was used to threshold the analysis. The color bar represents the F score. Yellow presents a high F score. Abbreviations: HC, healthy control; PD, Parkinson’s disease; WO, wearing-off; GM, grey matter; L, left, R, right

Compared with HC group, PD patients without WO showed increased GM volumes in left cerebellum (cerebellum anterior lobe, lobules IV–VI), bilateral frontal cortex (left middle frontal gyrus, left orbital part of middle frontal gyrus, left orbital part of inferior frontal gyrus, left triangular and opercular part of inferior frontal gyrus, and right superior frontal gyrus), parietal lobe (right precuneus, inferior and superior parietal gyrus), bilateral temporal cortex (superior temporal gyrus, left middle temporal gyrus, and right Heschl’s gyrus), right precuneus, right superior parietal gyrus, right lingual gyrus and middle occipital gyrus. Results are illustrated in Table 3 and Fig. 2.

**Table 3:** Differences in grey matter volume between HC and PD-nWO groups

Contrast	No. of clusters	L/R	Anatomical region	Voxels	Peak MNI coordinates			*T*-value
					X	Y	Z	
PD-nWO > HC	2	L	Cerebellar anterior lobe, lobules IV–VI	185	−30	−18	−48	3.38
	4	R	Lingual gyrus, middle occipital gyrus, calcarine, cuneus	149	21	−96	−18	2.93
	5	L	Orbital of inferior frontal gyrus, orbital of middle frontal gyrus	48	−33	33	−21	2.64
	6	R	Superior Frontal Gyrus	33	21	72	6	2.59
	7	L	Middle frontal gyrus, triangular inferior frontal gyrus, opercular part of inferior frontal gyrus	168	−54	24	30	2.72
	8	R	Superior temporal gyrus, Heschl’s gyrus	28	54	−12	0	2.46
	9	L	Superior temporal gyrus, medial temporal gyrus	36	−63	−18	6	2.54
	10	L	Inferior parietal lobe, frontal Lobe	71	−36	−36	30	3.1
	12	R	Precuneus, superior parietal gyrus	24	9	−78	54	2.54
PD-nWO < HC	–	–	–	–	–	–	–	–

MNI, Montreal Neurological Institute space; The X, Y, and Z coordinates refer to the anatomical location of brain areas

*PD*, Parkinson's disease; *HC*, healthy control group; *WO*, wearing-off; *L*, left; *R*, right

### Comparison of GM changes between PD-WO and HC groups

**Fig. 3:**
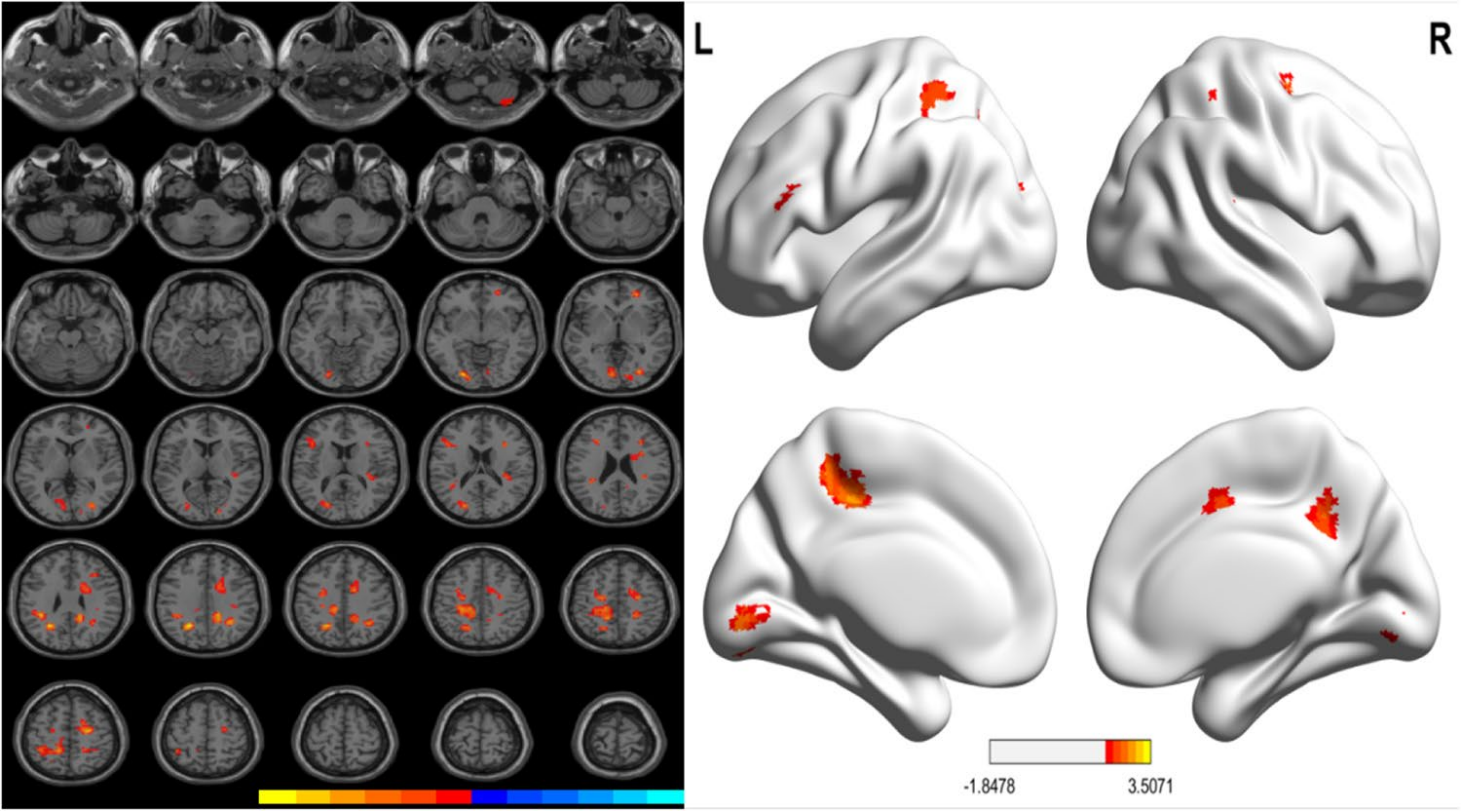
Significant differences in GM volumes between the HC and PD-WO groups. Correction for multiple comparisons (FWE, *P* < 0.05) was used to threshold the analysis. The color bar represents the F score. Yellow presents a high F score. Abbreviations: HC, healthy control; PD, Parkinson’s disease; WO, wearing-off; GM, grey matter; L, left, R, right

Compared with the HC group, PD patients with WO showed increased GM volume in the right cerebellum posterior lobe and lobule III, bilateral lingual gyrus, calcarine cortex, occipital lobe, parietal lobe (postcentral gyrus, precuneus, left inferior and superior parietal gyrus), frontal gyrus (left precentral gyrus, right supplementary motor area, inferior and superior frontal gyrus), left median cingulate and paracingulate gyri. These results were presented in Table 4 and Fig. 3.

**Table 4:** Differences in grey matter volume between HC and PD-WO groups

Contrast	No. of clusters	L/R	Anatomical region	Voxels	Peak MNI coordinates			*T*-value
					X	Y	Z	
PD-WO > HC	1	R	Cerebellar posterior lobe, lobule III	26	36	−75	−57	2.32
	2	L	Lingual gyrus, calcarine, inferior occipital gyrus, fusiform gyrus	97	−24	−81	−6	3.26
	3	R	Calcarine, lingual gyrus, occipital lobe	29	15	−90	9	2.28
	4	R	Orbital part of superior frontal gyrus, middle frontal gyrus	33	24	48	0	2.79
	5	R	Middle occipital gyrus	41	27	−78	0	2.81
	6	L	Middle occipital gyrus, cuneus, superior occipital gyrus	57	−21	−78	18	2.86
	8	L	Triangular part of inferior frontal gyrus	59	−42	21	18	2.48
	10	L	Inferior parietal lobule, supramarginal gyrus	65	−39	−39	30	3.09
	11	R	Cingulate gyrus, limbic lobe, superior frontal gyrus, median cingulate and paracingulate gyri, supplementary motor area	278	24	−12	54	3.02
	12	R	Parietal lobe, precuneus	100	15	−39	33	3.04
	13	L	Middle occipital gyrus, precuneus, superior parietal gyrus	110	−24	−57	33	3.51
	15	L	Inferior parietal lobule, median cingulate and paracingulate gyri, postcentral gyrus	334	−15	−45	51	3.04
	16	L	Precentral gyrus	77	−21	−12	48	2.62
	17	R	Postcentral gyrus	43	21	−42	51	2.70

MNI, Montreal Neurological Institute space; The X, Y, and Z coordinates refer to the anatomical location of brain areas

*PD*, Parkinson's disease; *HC*, healthy control group; *WO*, wearing-off; *L*, left; *R*, right

### Comparison of GM changes between PD-WO and PD-nWO groups

**Fig. 4:**
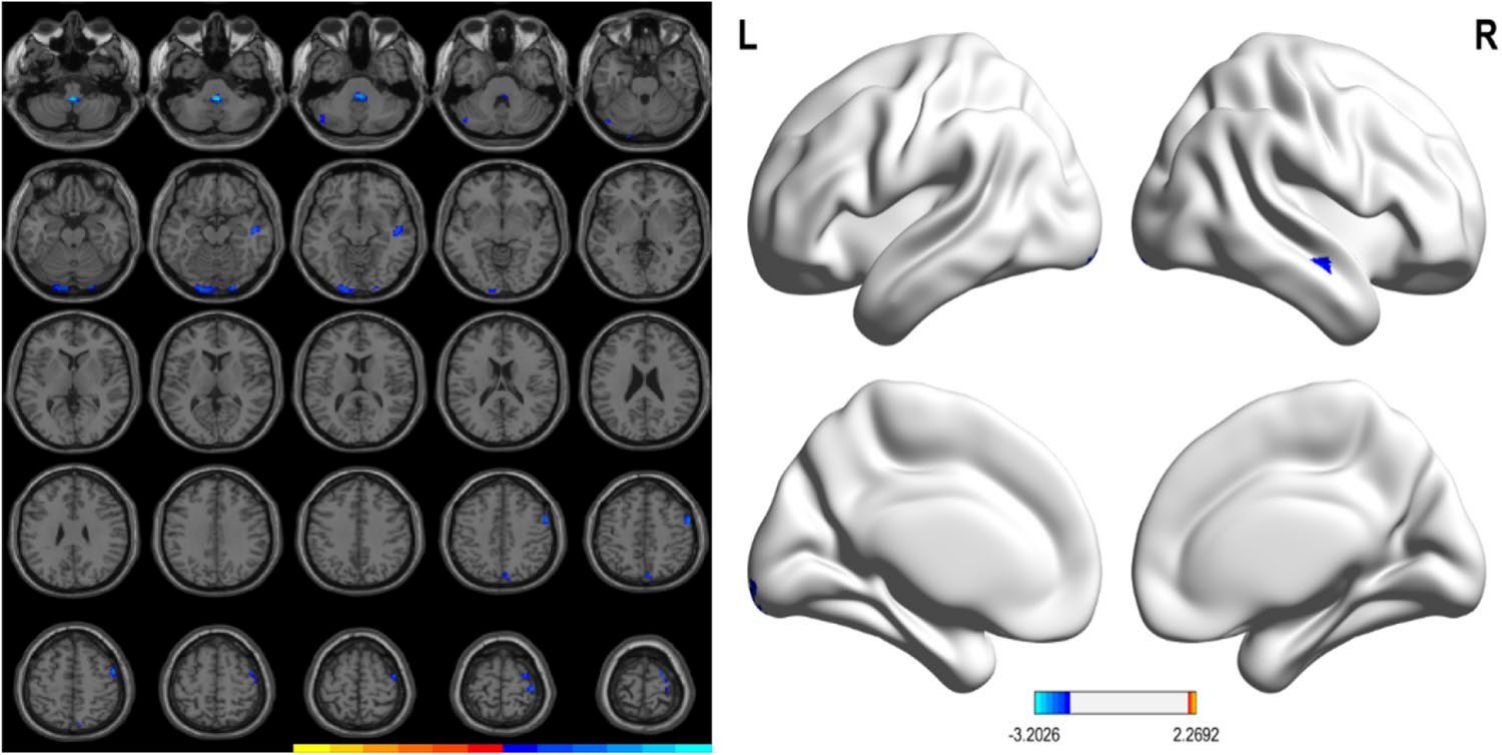
Significant reductions in GM volumes between the PD-nWO and PD-WO groups. Correction for multiple comparisons (FWE, *P* < 0.05) was used to threshold the analysis. The color bar represents the F score. Yellow presents a high F score. Abbreviations: HC, healthy control; PD, Parkinson’s disease; WO, wearing-off; GM, grey matter; L, left, R, right

The decreased GM volumes in PD patients with WO were observed in frontal cortex (right middle and superior frontal gyrus, precentral gyrus), parietal lobe (right precuneus, superior parietal gyrus, postcentral gyrus), right temporal cortex (superior temporal gyrus, middle temporal gyrus), bilateral lingual gyrus and inferior occipital gyrus, and left cerebellum Crus I and posterior lobe. These results were summarized in Table 5 and Fig. 4.

**Table 5:** Differences in grey matter volume between PD-nWO group and PD-WO group

Contrast	No. of clusters	L/R	Anatomical region	Voxels	Peak MNI coordinates			*T*-value
					X	Y	Z	
PD-nWO > PD-WO	2	L	Cerebellum Crus I, cerebellum posterior lobe	33	−48	−75	−27	−2.30
	3	L	Inferior occipital gyrus, lingual gyrus, calcarine	115	−9	−99	−18	−2.46
	4	R	Inferior occipital gyrus, lingual gyrus	24	21	−99	−18	−2.46
	5	R	Medial temporal gyrus, superior temporal gyrus	38	48	−15	−15	−2.56
	6	R	Precuneus, superior parietal gyrus	21	6	−78	51	−2.31
	7	R	Middle frontal gyrus, precentral gyrus	66	54	0	51	−2.71
	8	R	Precentral gyrus, superior frontal gyrus, postcentral gyrus	57	36	−24	72	−2.64
PD-nWO < PD-WO	–	–	–	–	–	–	–	–

MNI, Montreal Neurological Institute space; The X, Y, and Z coordinates refer to the anatomical location of brain areas

*PD*, Parkinson's disease; *HC*, healthy control group; *WO*, wearing-off; *L*, left; *R*, right

### Correlation analysis

**Fig. 5:**
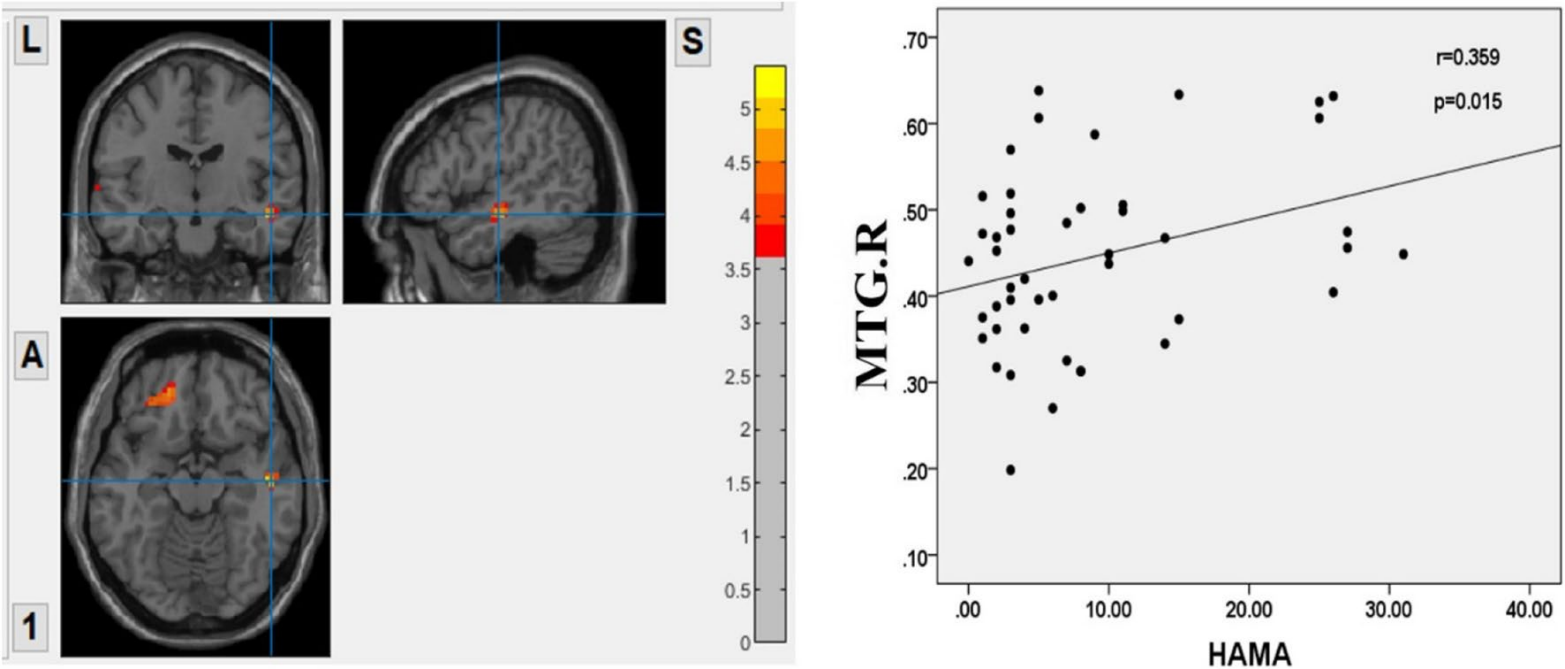
Correlation between the GM volumes of the right middle temporal gyrus (MTG.R) showing difference and HAMA scores in PD patients without wearing-off. The GM volumes of MTG.R was positively correlated with HAMA scores (*r* = 0.359, *P* = 0.015). The color bar represents the F score. Yellow presents a high F score. Abbreviations: HC, healthy control; PD, Parkinson’s disease; WO, wearing-off; GM, grey matter; HAMA, Hamilton Anxiety Scale; L, left, R, right

We performed Pearson correlation analyses between the significantly altered GM volumes and clinical performance. Significant positive correlation was found between HAMA and GM volume in the right middle temporal gyrus in the PD-nWO group (*r* = 0.359, *P* = 0.015). The correlation is presented in Fig. 5.

## Discussion

In the present study, we aimed to analyze the GM changes in whole brain in PD patients with wearing-off using VBM. Our results found significant reductions in GM volumes of PD patients with wearing-off in frontal cortex, temporal cortex, lingual gyrus and inferior occipital gyrus compared with those without wearing-off. Our findings suggest that GM abnormalities might be associated with the severity of neuropsychological symptoms and motor symptoms deterioration in PD patients with wearing-off.

In the present study, the findings of GM volume changes in PD patients were not wholly consistent with previous studies. The results difference of these studies may be due to the heterogeneity of the age, education, disease duration and severity of PD patients. Interpretation should be cautious although several covariates were included in this study to control for potential confounding effects.

Although, the pathogenesis of wearing-off is not well understood, it may be attributable to the degree of nigrostriatal neurodegeneration and synaptic abnormalities in striatal neurons related to chronic levodopa therapy (Chung et al., 2018; Freitas et al., 2017). Levodopa can increase the number of synapses, improve the white matter integrity and gray matter density in the hippocampus in rats (Wang et al., 2020). Chronic levodopa administration affects the function of dopaminergic pathways in brain regions in PD patients or healthy individuals (Hershey et al., 2003; Salgado-Pineda et al., 2006). Studies in structural MRI have found that the frontal cortex was overactive in levodopa-induced dyskinesias patients. Their data suggested that chronic levodopa administration may cause functional abnormalities in specific brain areas (Cerasa et al., 2012). Ballarini et al. demonstrated that a reduced GM density was associated with a weaker response to dopaminergic therapy in PD patients (Ballarini et al., 2019). In this study, GM volumes in partial cortical region were increased in PD patients when compared with HC, while the decreased GM volumes in other cortical region did not reach a significant difference. The main cause might be that the decreased cluster size was small. Consistent with our work, previous studies reported increased GM volume in occipital, limbic and paralimbic regions in PD patients, which could represent a compensatory mechanism to impaired brain function in PD (Jia et al., 2015; Pagonabarraga et al., 2014). Furthermore, the GM volumes in cortical regions were reduced in PD patients with wearing-off when compared with those without wearing-off. These findings indicating that a reduced GM volume in the PD patients with wearing-off was associated with a weak response to dopaminergic therapy and progression. These results might help us to better understand the degeneration process of PD.

Many studies showed cortical reduction in GM volumes in PD. GM atrophy of the inferior frontal gyrus was significantly associated with attention cognitive deficit (Yu et al., 2021). With the progress of PD, neocortex areas including limbic and paralimbic system, frontal, temporal, and occipital regions were gradually atrophy (Zhang et al*.*, 2015). In accordance with previous study, we also found GM atrophy in frontal, temporal and occipital cortex in PD patients with wearing-off. Subcortical parts of the brain by VBM analysis were also observed in previous studies. Cerasa et al. reported that PD patients have increased volume in basal ganglia and thalamus (Cerasa et al., 2011). It has also been reported that volumes reduction in the caudate nucleus and thalamus was observed in PD, which may be an early phenomenon of disease progression (Lee et al., 2011). In this study, only cortical GM volumes were analyzed by VBM, it is not clear the changes in subcortical areas in PD patients with wearing-off. In the future, we need to detect the links between cortical and subcortical areas in wearing-off.

Evidence shows that motor cortex dysfunction is an important component of PD pathophysiology (Lindenbach & Bishop, 2013). Previous studies reported that GM volumes in the inferior frontal gyrus was increased in PD patients with dyskinetic (Cerasa et al*.*, 2011, 2013). The reduction of GM volume in the right inferior frontal gyrus was linked to the higher risk of falling (Cheng et al., 2020). The decrease in supplementary motor area (SMA) has been reported in previous study (Jubault et al., 2011). However, some studies have not observed GM changes in the motor cortex in PD (Cerasa et al*.*, 2011; Lindenbach & Bishop, 2013). The increased GM volume of precentral gyrus was more likely to develop dyskinesia in PD (Zhi et al., 2019). In our study, GM volume in SMA was increased in PD patients with wearing-off compared with controls, indicating that the increased GM volume of SMA might be caused by aberrant plasticity and chronic dopaminergic therapy. In addition, decreased GM volume in the precentral gyrus was observed in PD patients with wearing-off compared with those without wearing-off. The reduction in the frontal gyrus and precentral gyrus suggested that GM atrophy in the motor cortex might be associated with motor symptoms deterioration in PD patients with wearing-off.

PD-related changes in the limbic system are linked not only to alterations in emotion processing but also to a more extensive symptom complex including cognitive impairment, sleep disorders, and motor dysfunction. Some analysis revealed GM atrophy in several brain regions, including frontal gyrus, orbitofrontal, temporal lobe, precuneus, and cerebellar were linked to neuropsychological performances in PD (Chen et al., 2019; Donzuso et al., 2021; Gao et al., 2017). The lingual gyrus has been linked to visual processing activities. A recent study showed that GM atrophy in the left lingual gyrus in PD patients with dementia, especially associated with letters and logical analysis dysfunction (Nyatega et al., 2022). Other study reported the reduction of GM volumes in lingual gyrus could be associated with freezing of gait in PD (Tessitore et al., 2012). In line with other studies, GM atrophy in the lingual gyrus was also found in PD patients with wearing-off, suggesting that the presence of freezing of gait has been associated with atrophy of the lingual gyrus in mid- and late-stage of PD.

However, our study reported increased GM volume in cerebellar Crus I in PD patients with wearing-off compared to the controls. The GM alterations in the left medial and right cerebellar Crus I may be related to Cognitive and executive deficits in PD (Dirnberger & Jahanshahi, 2013). PD patients suffer from depression and tremor may be associated with the abnormal connectivity within the pathological interaction between the basal ganglia and cerebello-thalamo-cortical circuit (Lewis et al., 2011). Zeng et al. found significant decreased in GM volume in the cerebellar Crus I, Vermis III and VIII in PD patients, demonstrated that cerebellar GM atrophy may also be involved in PD cognitive impairment severity (Zeng et al., 2017). For the right cerebellar Crus I, in addition to a possibility of pathophysiological change, the increased GM volume may be a compensation for basal ganglia dysfunction to maintain motor function at a near normal level (Lewis et al., 2011).

The MMSE and MoCA are the common methods to detect cognitive impairment in clinical (Lim & Loo, 2018). In our study, the Chinese version of MoCA test was administered in all participants. We also noticed that the mean MoCA scores was significantly lower in three groups in our results. According previous study, MoCA score is highly dependent on cultural background of the subject. Educational level was found to be the strongest unpredictable factor affecting MoCA test scores (Lu et al*.*, 2011; Jia et al., 2021). At present, the size of the elderly population in China is growing, and the education level of them is relatively low. The lack or limitation of education may affect the accuracy of the evaluation in MoCA (Wu et al., 2023). According to Chinese MoCA norms, mild cognitive impairment (MCI) was identified ≤ 13 for illiterate individuals, ≤ 19 for those with 1–6 years of education, and ≤ 24 for those with 7 or more years of education(Lu et al*.*, 2011). However, the elderly and lower education level individuals may struggle to complete the full MoCA. As a result, the revised version of MoCA basic (MoCA-B) test may suitably screening tool for those population (Julayanont et al., 2015). Besides those mentioned above, the MoCA may possibly give rise to a higher false positive rate than the MMSE (Aiello et al., 2022; Larner, 2012).

## Limitations

There are some limitations that should be considered in the present study. First, the small sample size of our study should be considered when interpreting these findings. Therefore, our study needs a larger sample size and longitudinal follow-up for further investigation. Second, this study was confined to investigate alterations in the GM volume of the whole brain, and the mechanism of wearing-off should be further explored by other methods such as diffusion tensor imaging (DTI) and rest-state MRI. Third, selection bias in our sample may have affected the results of these neuropsychological tests. The data of our research were mainly based on low-level education and older group of suburban residents. Finally, as this is a cross-sectional investigation, a prospective study would be better to observe structural abnormalities dynamically in brain.

## Conclusions

In summary, this was the first study to detect GM volumes in subjects with wearing-off compared to those without wearing-off and healthy subjects using VBM. Our study confirmed the existence of a general cortical atrophic process following the progression of wearing-off in PD, described as a decreased GM volume in cortical regions and appear to be related to disease-related structures, although there was no significant correlation between altered GM volumes and clinical performance in PD patients with wearing-off. These findings have promoted our understanding of the underlying neural mechanism divergence from a network perspective during PD progression. VBM was more sensitive compared with conventional MRI in monitoring brain changes and may be used in the long-term follow-up of PD patients with wearing-off. Future studies examining mechanisms of wearing-off will need to be confirmed using other fMRI modalities.

## Data Availability

Data sharing is allowed for other investigations to replicate the results.
